# Assessing Socioeconomic and Environmental Vulnerability and Awareness of Zoonotic Diseases From a One Health Perspective: A Cross-Sectional Study in a Rural Population in Tamil Nadu, India

**DOI:** 10.7759/cureus.81198

**Published:** 2025-03-25

**Authors:** Trupti Bodhare, Samir Bele, Santha Sheela Kumari, Ramji M, Shalini V, Bharath Rajh, Hariharan V

**Affiliations:** 1 Community and Family Medicine, All India Institute of Medical Sciences, Madurai, IND; 2 Community Medicine, Velammal Medical College Hospital and Research Institute, Madurai, IND

**Keywords:** attitude, knowledge, one health, practices, vulnerability, zoonotic diseases

## Abstract

Background: The interconnection between human, animal, and ecosystem health is crucial, particularly in rural areas, where several socio-economic and environmental risk factors heighten vulnerability to zoonotic diseases. This study aims to assess the vulnerability as well as knowledge and practices concerning zoonosis from a One Health perspective.

Methodology: A cross-sectional study was carried out in rural areas of Madurai district, Tamil Nadu, from July to December 2024, selected by simple random sampling. The socio-demographic and ecological risk factors, knowledge, attitudes, and practices regarding zoonosis were assessed utilizing a validated semi-structured questionnaire among 408 participants. Microsoft Excel (Microsoft, Redmond, WA, USA) and R programming (version 4.4.3; R Foundation for Statistical Computing, Vienna, Austria) were used for analyzing the data.

Results: The study sample, comprising 238 (58.3%) males, had a mean age of 43.32 years, with 46 (11.3%) being illiterate. Around 134 (32.84%) belonged to the lower middle class, with 50 (12.3%) worked in agriculture, and 118 (28.9%) were unemployed. Around 96 (23.53%) of the population lived in kutcha houses, 151 (37.01%) reported dampness, and 253 (62.01%) reported overcrowding. Environmental issues include mosquito breeding in 279 (68.38%), open drains in 268 (65.69%), stray animals in 308 (75.49%), and rat infestations in 155 (37.99%) of communities. Around 321 (79%) of individuals demonstrated good knowledge, while only 96 (24%) exhibited a positive attitude and 107 (26%) engaged in good practices. We noted a negative attitude and poor practices among 120 (29%) and 170 (42%), respectively. Individuals aged 41 years and older (p=0.02) and pet owners (p=0.01) demonstrated a higher level of knowledge.

Conclusions: This study emphasizes the importance of addressing vulnerabilities in socially and ecologically disadvantaged populations to prevent zoonotic diseases. It identifies a gap between knowledge and practical application, advocating for behavior change for better rural health outcomes.

## Introduction

The health of humans, animals, and the ecosystem are closely interlinked. Changes in these relationships can increase the risk of emergence and spreading of new human and animal diseases as well as risk of outbreak and persistence of endemic zoonosis [1].

Zoonotic diseases significantly impact global healthcare, especially in underdeveloped nations. Around 60% of infectious diseases are of animal origin, and 75% of emerging diseases are zoonotic. Threats such as climate change, environmental degradation, zoonotic diseases, antimicrobial resistance, and food safety hazards are intricately linked to human activities that impact animals, plants, and the environment [2,3].

In India, where 70% of the population resides in rural areas, only a small fraction has access to primary healthcare centres, subcentres, or hospitals. With the growing population and limited resources, people are forced to live in closer proximity to animals, which increases their vulnerability to exposure to pathogens and toxins. Several socio-economic and environmental risk factors, like cultural practices, animal husbandry, agricultural practices, home sanitation, climate, and geography play an important role in the occurrence of zoonotic diseases. Lack of understanding about disease transmission, prevention, and control practices poses a high risk for the incidence of zoonotic illnesses in rural areas [4,5].

Vulnerability in public health arises from a complex interplay of physical, social, economic, and environmental factors that increase susceptibility to hazards. Access to safe drinking water, sanitation, hygiene (WASH), and healthcare are fundamental determinants of health, yet millions worldwide face critical deficiencies in these areas. Vulnerability assessments help identify vulnerable populations and help policymakers and communities in responding to and minimizing infectious threats [6].

Studies done in the Indian subcontinent highlight a significant lack of knowledge and awareness regarding proper hygiene and health practices in relation to animal interactions and food consumption. This ignorance contributes to various health risks, including exposure to several zoonotic diseases. The prevalent issues include failure to seek medical attention, reliance on traditional medicine, and unhygienic behaviors such as consuming raw or contaminated food and inadequate sanitation practices. Addressing these gaps by enhancing community knowledge and awareness about preventive measures and improving health practices is crucial to minimizing the risks associated with animal-related diseases and preventing disease spillovers [7].

Addressing these challenges requires a multidisciplinary approach that includes public health interventions, improved infrastructure, and policy reforms. Strengthening WASH services, enhancing healthcare accessibility, and adopting a One Health perspective can mitigate vulnerability, reduce disease burden, and promote health equity. This approach aims to balance and optimize the health of people, animals, and ecosystems, making it easier for people to understand co-benefits, risks, trade-offs, and opportunities for equitable solutions. However, successful implementation of One Health is hindered in developing and underdeveloped countries, particularly in rural areas where people are vulnerable to zoonotic diseases due to factors like animal trade, agriculture, and human-animal interactions [3,7]. There is a notable lack of empirical research regarding the vulnerability, particularly within the rural South Indian community, to emerging zoonoses and their knowledge and preventive practices regarding disease transmission [7,8].

Hence the current study is planned to assess the vulnerability of the rural population in the context of their socio-demographic and environmental characteristics, human-animal-environmental interface, knowledge about zoonosis and One Health issues, health practices related to sanitation, hygiene, and other risk factors for zoonosis.

## Materials and methods

Study design and setting

A descriptive, cross-sectional study was conducted in the rural areas of Madurai district, Tamil Nadu, over a six-month period from July to December 2024.

Sampling technique

A community-based study was conducted in the rural area of Madurai district. A multistage random sampling technique was used. The Madurai district comprises 11 taluks, with one village randomly selected from each taluk using a simple random sampling technique via a lottery method. Households in each village were selected through a simple random sampling method. From each selected household, one available adult selected randomly was interviewed with a validated semi-structured questionnaire. The total sample size was evenly allocated across the 11 villages.

Pilot study

The pilot study played a crucial role in validating the questionnaire and assessing its reliability using Cronbach’s alpha, which was found to be 0.8, ensuring the reliability of the study instrument. A total of 30 participants were involved in the pilot study.

Sample size

For the study, the necessary sample size is determined based on the following assumptions: The desired confidence level is set at 95%, with a margin of error of 5%. A pilot study indicated that the knowledge of zoonosis within the population stands at 52%. Using the formula n=Z^2^P(1-P)/d^2^, the minimum required sample size was 383.

Inclusion and exclusion criteria

The study included adults who have been residents of the selected villages for a minimum of six months, ensuring a stable and relevant participant base. However, individuals with severe cognitive impairments or those with significant hearing or vision impairments that could hinder their ability to engage in the study had been excluded from the study.

Data collection

The study involved participants who provided written informed consent and completed a validated questionnaire. A semi-structured questionnaire was used to collect information about the socio-demographic, household and environmental characteristics of the participants. The questionnaire assessed knowledge, attitude and practices regarding zoonosis, risk factors for zoonosis such as household practices related to sanitation, hygiene, and environmental contaminants, contact with pets, breed animals or wild animals etc. Knowledge, attitude and practices about One Health was also assessed in the context of environmental health and its linkage with animal and human health.

The pilot study played a crucial role in validating the questionnaire and assessing its reliability using Cronbach’s alpha, which was found to be 0.8, ensuring the reliability of the study instrument. A total of 30 participants were involved in the pilot study.

Data analysis

The collected data were entered into Microsoft Excel (Microsoft, Redmond, WA, USA) and analyzed using R programming (version 4.4.3; R Foundation for Statistical Computing, Vienna, Austria). Descriptive statistics, including means and standard deviations were employed to characterize continuous variables, whereas proportions were utilized for categorical variables. To determine the participants' socioeconomic status, we used B. G. Prasad's revised classification of socioeconomic status for the year 2024 [9]. To evaluate knowledge, attitudes, and practices, responses were scored as 1 for "Yes" (affirmative response) and 0 for "No" (negative response), with the total score derived from the aggregation of positive replies. The cumulative knowledge, attitude and practices scores were subsequently transformed into percentages, with 100% signifying complete concordance with the intended knowledge, attitudes, and practices. According to these percentage values, all three domains (knowledge, attitude, and practices) were uniformly classified as low (0-49%), moderate (50-79%), and high (80-100%). The association between variables was determined using chi-square/Fisher's exact tests. A p-value below 0.05 was regarded as statistically significant.

Ethical consideration

The study adhered to ethical standards by ensuring participant anonymity, obtaining informed written consent, and securing clearance from the Institutional Ethics Committee of the institute (IEC No: VMCIEC/066/2024).

## Results

Table 1 describes 408 people who participated in the study. The study sample consists of individuals with a mean age of 43.32 years (±1.42 SD). Among them, 238 (58.3%) were male, and 170 (41.7%) were female. Regarding education, the majority (237, 58.1%) had completed schooling, while 46 (11.3%) were illiterate. In terms of occupation, 240 (58.8%) were engaged in non-agricultural jobs, while 50 (12.3%) worked in agriculture, and 118 (28.9%) were unemployed. The average monthly income of the participants was Rs.23,517.16 (±Rs.15,453.49 SD). Socioeconomic status distribution shows that most participants belong to the lower middle class (134, 32.84%) followed by the middle class (114, 27.94%). 

**Table 1:** Socio-demographic characteristics of the participants N: Number of participants, SD: Standard Deviation

Variable	Categories	N	%
Age (Mean ± SD)		43.32 ± 1.42	
Gender	Male	238	58.3
	Female	170	41.7
Education	Illiterate	46	11.3
	Schooling	237	58.1
	Graduate	111	27.2
	Professional Degree	14	3.4
Occupation	Unemployed	118	28.9
	Agriculture	50	12.3
	Non-Agriculture	240	58.8
Income (Mean ± SD)		23517.16 ± 15453.49	
Socioeconomic status	Upper class	20	4.91
	Upper middle class	83	20.34
	Middle class	114	27.94
	Lower middle class	134	32.84
	Lower class	57	13.97

Table 2 outlines the details of the participants' living environmental conditions. The majority lived in pucca dwellings (275, 67.4%), followed by kutcha houses (96, 23.53%). Around 151 (37.01%) households reported dampness, while 253 (62.01%) of participants reported overcrowding. Around 226 (55.39%) participants were using some form of water purification method at home. About 279 (68.38%) participants responded that mosquito breeding sites were present around the households, while 308 (75.49%) participants reported stray animals in the locality. Around 268 (65.69%) the participants reported open drains in the locality, while 155 (37.99%) reported rat infestation. A total of 328 (80.39%) people had access to common trash disposal outlets. A total of 57 (13.97%) had suffered animal-related injuries. Around 76 (18.6%) of the population owned pets, 59 (14.5%) owned breeds, and 18 (4.4%) of individuals owned both breed animals as well as pets.

**Table 2:** Housing and environmental conditions of the participants N: Number of participants, SD: Standard Deviation

Variable	Categories	N	%
Housing type	Pucca	275	67.40
	Semi pucca	37	9.07
	Kutcha	96	23.53
Presence of dampness in the house	Yes	151	37.01
	No	257	62.99
Overcrowding	Present	253	62.01
	Absent	155	37.99
Availability of purified water at home	Yes	226	55.39
	No	182	44.61
Presence of mosquito breed / water shed areas in surroundings	Yes	279	68.38
	No	129	31.62
Existence of stray animals in the locality	Yes	308	75.49
	No	100	24.51
Presence of open drains in the locality	Yes	268	65.69
	No	140	34.31
Presence of rodent infestation	Yes	155	37.99
	No	253	62.01
Availability of common waste disposal points	Yes	328	80.39
	No	80	19.61
History of injury inflicted by the animal in the past	Yes	57	13.97
	No	351	86.03
Ownership	No	255	62.5
	Pet	76	18.6
	Breed	59	14.5
	Breed and pet	18	4.4
Time travel to reach to nearby health facilities (in minutes) (Mean ± SD)		13.44 ± 7.55	

Table 3 indicates that the highest level of knowledge was regarding the importance of using disinfectants when disposing of dead or infected animals (343, 84.1%), followed closely by the understanding that cutting forests can damage the environment and increase illness (340, 83.3%). On the other hand, 338 (82.8%) participants were aware that humans can contract diseases from animals. Nearly 131 participants (32.1%) expressed concern about diseases transmitted by animals, while 130 individuals (31.9%) stated that it is acceptable to use wild animals for traditional medicine or beauty products. Around 343 (84.1%) expressed their interest in contributing to the improvement of environmental hygiene and sanitation. A total of 61 participants (15%) were identified consuming raw milk, while another 61 (15%) received health education regarding environmental protection. Only 53 (13%) have engaged in environmental protection activities.

**Table 3:** Participants knowledge attitude and practices regarding zoonotic diseases, and environmental health N: Number of participants

Knowledge			
Variables	Categories	N	%
Human can acquire a disease from animals	Yes	338	82.8
	No	70	17.2
Proximity to animals can elevate the risk of disease transmission	Yes	316	77.5
	No	92	22.5
Wild animals can infect domesticated animals	Yes	336	82.4
	No	72	17.6
Disinfectant chemicals should be used when handling dead or infected animals	Yes	343	84.1
	No	65	15.9
Human, animal and environmental health are linked	Yes	339	83.1
	No	69	16.9
Polluting the air, water or land with chemicals may cause illness	Yes	332	81.4
	No	76	18.6
Cutting forests can damage environment and increase illness	Yes	340	83.3
	No	68	16.7
Improper waste management, can harm the environment	Yes	337	82.6
	No	71	17.4
Various measures can prevent acquiring disease from animals	Yes	298	73
	No	110	27
Vaccination protects animals from disease	Yes	338	82.8
	No	70	17.2
Attitude			
Pet and livestock ownership offers benefits	Yes	182	44.6
	No	226	55.4
I am concerned about diseases caught from animals	Yes	131	32.1
	No	277	67.9
It's okay to use wild animals for traditional medicine or beauty products	Yes	130	31.9
	No	278	68.1
It’s everyone’s duty to keep the environment safe	Yes	282	69.1
	No	126	30.9
There should be environmental regulations in reducing exposure to harmful toxins	Yes	269	65.9
	No	139	34.1
Interested in contributing to the improvement of environmental hygiene and sanitation	Yes	343	84.1
	No	65	15.9
Practices			
Drink raw milk	Yes	61	15
	No	347	85
Wash hands with soap and water after having contact with animals	Yes	261	64
	No	147	36
Walk outside without footwear	Yes	94	23
	No	314	77
Received health education on environmental protection	Yes	61	15
	No	347	85
Participated in any activities that promote environmental protection	Yes	53	13
	No	355	87

**Figure 1:**
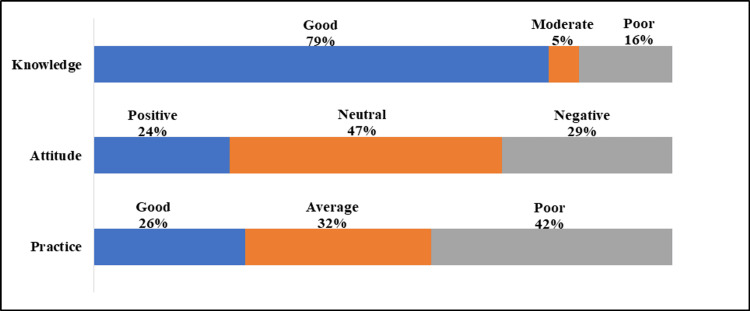
Knowledge, attitude and practices scores among the participants

Figure 1 indicates that a total of 321 (79%) individuals demonstrated good knowledge; however, only 96 (24%) exhibited a positive attitude, and 107 (26%) engaged in good practices. We noted a negative attitude and inadequate practices among 120 (29%) and 170 (42%), respectively. A strong positive correlation was found between practices and attitude (r=0.989, p=0.001).

Table 4 presents the association between knowledge, attitude, and practice scores and the socio-demographic characteristics of the participants. A greater level of knowledge was noted in the age groups of 41 years and older (p=0.02), as well as among pet owners (p=0.01). We observed no significant association between knowledge, attitude, and practices and other socio-demographic characteristics like education, occupation, and socioeconomic status.

**Table 4:** Association of knowledge, attitude and practices with socio-demographic characteristics L: Low, M: Moderate, H: High

Variables	Categories	Knowledge			Attitude			Practices		
		L	M	H	L	M	H	L	M	H
Age	Below 20	2	0	1	1	2	0	1	2	0
	21 to 40	22	9	77	42	33	33	38	34	36
	41 and above	42	12	243	127	96	74	118	103	76
	p value	0.02			0.38			0.51		
Education	Illiterate	4	5	37	17	16	13	14	19	13
	Schooling	39	9	189	101	77	59	96	81	60
	Diploma	1	1	1	0	2	1	0	2	1
	Graduate	20	4	84	44	30	34	41	30	37
	Professional degree	2	2	10	8	6	0	6	7	1
	p value	0.07			0.23			0.28		
Occupation	Unemployed	23	8	87	36	48	34	42	42	34
	Agriculture	5	2	43	20	13	17	20	17	13
	Non-Agriculture	38	11	191	101	78	61	108	76	56
	p value	0.46			0.16			0.29		
Socio economic status	Upper class	4	3	13	7	6	7	6	7	7
	Upper middle class	11	3	69	35	30	18	33	31	19
	Middle class	21	6	87	51	33	30	48	34	32
	Lower middle class	21	7	106	56	44	34	49	49	36
	Lower class	9	2	46	21	18	18	21	18	18
	p value	0.62			0.89			0.90		
Ownership	No	33	14	208	102	86	67	97	86	72
	Pet	9	4	63	30	24	22	27	27	22
	Breed	17	3	39	28	18	13	25	21	13
	Breed and pet	7	0	11	10	3	5	8	5	5
	p value	0.01			0.95			0.71		

## Discussion

The current study aimed to assess the vulnerability of rural populations to zoonotic diseases using the One Health lens. It focused on identifying factors contributing to vulnerability, including healthcare accessibility, livestock, pet ownership, housing, and environmental hazards. While several studies have been conducted on individual zoonotic diseases; the current study also explored the knowledge about the role of the environment in animal and human health and its linkage and importance.

The current study revealed that 58.1% of individuals have completed schooling, with 11.3% being illiterate. Occupationally, a substantial portion of the population was unemployed (28.9%) while 12.3% work in agriculture with most participants falling within the lower middle class (32.84%). Asaaga et al. found that 29.7% of household heads in the Western Ghats of India lack formal education and 27.9% had secondary education. Over half of the families depend on agriculture, and many live below the poverty line, making them vulnerable to zoonotic illnesses [10].

In our study, a significant proportion of the participants faced ecological vulnerability, such as 23.53% of participants living in kutcha houses, 37.01% experiencing dampness, and 62.01% experiencing overcrowding. Mosquito breeding grounds were present in 68.38% of areas, and 75.49% have stray animals. 65.69% of communities reported open drains, while 37.99% reported rat infestation. 13.97% of the population reported animal-related injuries. Around 18.6% were having close contact with pets, 14.5% with breeds. A study conducted by Kim et al. showed that 46% of the households assessed were at moderate to high risk for exposure to zoonotic pathogens and environmental contaminants [11]. The scoping review by Durrance-Bagale et al. identified significant drivers of zoonotic disease risk in the Indian subcontinent, including exposure to stray and domestic dogs and proximity to water bodies and poor housing, and overcrowding and shared water sources with livestock. Addressing these interconnected factors is crucial for mitigating zoonotic disease risks in the region [7].

Good level of knowledge (79%) of zoonotic diseases and environmental health was observed among participants with a strong awareness about transmission of a disease from animals to humans (82.8%), disinfectant use for prevention (84.1%), linkage of human, animal and environmental health (83.1%) and ill effects of deforestation (83.3%). The findings align with those of a study by Hundal et al., which indicated that around 84.8% and 92.4% of livestock farmers recognized the zoonotic nature of rabies and bird flu, respectively, and 55.6%, 67.2%, and 51.2% knew that zoonotic diseases can be transmitted from animals to people through contaminated milk, meat, or contact with infected animals [5]. A study conducted by Singh et al. showed that 80% of livestock farmers had heard the term 'zoonoses,' but only 40% were aware of the zoonotic nature of tuberculosis [4]. The study by Tae Youn Kim et al. conducted among a rural population found that 48% exhibited high One Health knowledge, while 18% demonstrated minimal understanding. Almost 97% acknowledged the risk of disease transmission from animals, plants, or the environment. Around 35% of respondents were aware of environmental contaminants impacting crops or animal drinking water [11]. Chinchwadkar et al. indicated that a significant majority (75%) of females with livestock in South-West Delhi demonstrated limited knowledge regarding specific zoonotic diseases [12]. According to a study by Sambath et al., 79.1% of community health workers and healthcare professionals in India knew about climate change and how it could affect their health by exposing them to disease-carrying agents [13]. The differences in knowledge levels can be attributed to diverse target participants, geographical and cultural variations, as well as the nature of the questions posed.

Risky practices related to zoonosis vary across the country. Hundal et al. found that 3.6% to 69.6% of participants consumed raw or unpasteurized animal products, with some using raw milk cream on skin cracks [5]. Biswas et al. reported only 24.07% of animal owners washed their hands with soap after handling animals, and 12.96% believed fresh raw milk is more nutritious than boiled milk [14]. The current study reveals concerning levels of poor practices and attitudes, with 42% and 29%, respectively. The study results may be attributed to the nature of the questions asked, such as whether participants have received health education on environmental protection or participated in environmental protection-promoting activities, which may depend on the performance of health and other sector activities in the area, leading to negative outcomes in the study findings. The strength, challenges, opportunities and threats analysis conducted by Bera et al. emphasized the critical need for enhanced stakeholder knowledge regarding the One Health Approach (OHA) across various disciplines. The author concluded that practical guidelines and cross-sectoral collaboration are crucial for OHA implementation [15].

While in the current study a significant majority demonstrated good knowledge, there is a notable gap in its application, with only 26% exhibiting good practices and 24% maintaining a positive attitude. Our findings are consistent with those of Chinchwadkar et al. who observed a significant gap in knowledge and practice [12]. This disparity underscores that while numerous individuals know the concepts, they struggle to implement them effectively in their daily lives. It indicates that the participants in the sample did not follow in practice compared to the knowledge which they have. However, the strong positive correlation between practice and attitude indicates that individuals with positive attitudes tend to have better practices.

Notably, we found no significant links with education, occupation, or socioeconomic status, but increasing age groups and pet ownership demonstrated higher knowledge levels. These results indicate that targeted educational interventions could improve knowledge and attitudes, especially among a general community and younger individuals. Our results are comparable with Yasobant et al. who demonstrated that age significantly influences knowledge of zoonoses, with each additional year correlating to a 0.3% increase in awareness [16]. Hundal et al. demonstrated that age, education, and herd size did not significantly influence the knowledge level and awareness of farmers regarding zoonotic diseases [5]. The findings indicate that various demographic and contextual factors may influence knowledge levels differently.

Limitations

This study presented a few limitations. The cross-sectional design limits the ability to draw causal inferences, and reliance on questionnaires may lead to potential biases. The limited sample size and concentration on a single district further restrict the generalizability of the findings. Future research should focus on exploring a wider array of vulnerability indicators.

## Conclusions

In conclusion, addressing the vulnerabilities of socially and ecologically disadvantaged populations is vital for preventing zoonotic diseases. The study highlights a critical gap between knowledge and its practical application, emphasizing the need for behavior change. Further interdisciplinary research is needed to understand risk perception of communities and to develop a holistic, multidisciplinary approach that considers the interplay between humans, animals, and the environment for improving rural health outcomes.

## Appendices

Appendix 1: Proforma

Section 1: Socio-Demographic Characteristics

What is your name? _____

What is your age (in years)?

Gender (as observed) -

Male                                  Female

Address of the residence -

What is your current marital status?

What is your highest level of education completed?

Which religion do you belong?

How long have you been living in this locality?

What is your current occupational status?

What is your total monthly family income?

What is the total number of family members in your household?

No of adults -                                No of Children -

Section 2: Environmental History

What is type of housing? (As observed)

Pucca

Semi pucca

Katcha

What is the total number of rooms in your house?

Is dampness present in your house?

Yes

No

What is the primary source of drinking water in your household?

Are you practicing any water purification method in your home?

Yes

No

Is there any presence of mosquito breeding/water shed areas in surroundings?

Yes

No

Don’t know

Is there stray animals present in the locality?

Yes

No

Don’t know

Are there any open drains present in the locality?

Yes

No

Don’t know

Is there any rodent infestation in your area?

Yes

No

Don’t know

Availability of common waste disposal points:

Yes

No

Don’t know

History of injury inflicted by an animal in the past:

Yes

No

Don’t know

History of seasonal illnesses in the past year:

Yes

No

Don’t know

Ownership of animals:

No ownership

Pet ownership

Breed ownership

Breed & pet ownership

How much time it takes to reach the nearest health facility? (in minutes)

Section 3: Knowledge

Can humans acquire diseases from animals?

Yes

No

Don’t know

Can proximity to animals elevate the risk of disease transmission?

Yes

No

Don’t know

Can wild animals infect domesticated animals?

Yes

No

Don’t know

Should we use disinfectant chemicals when handling dead or infected animals?

Yes

No

Don’t know

Are human, animal, and environmental health interconnected?

Yes

No

Don’t know

Can polluting air, water, or land with chemicals cause illness?

Yes

No

Don’t know

Can cutting forests damage the environment and increase illness?

Yes

No

Don’t know

Can improper waste management harm the environment?

Yes

No

Don’t know

Are there measures to prevent disease transmission from animals?

Yes

No

Don’t know

Can vaccination protect animals from disease?

Yes

No

Don’t know

Section 4: Attitude

Do pet and livestock ownership offer benefits?

Yes

No

Don’t know

Are you concerned about diseases caught from animals?

Yes

No

Don’t know

Is that okay to use wild animals for traditional medicine or beauty products?

Yes

No

Don’t know

Do you believe it's everyone’s duty to keep the environment safe?

Yes

No

Don’t know

Should there be rules about the environment to protect people from harmful toxins?

Yes

No

Don’t know

Are you interested in making a contribution to the improvement of environmental hygiene and sanitation?

Yes

No

Section 5: Practices

Do you drink raw milk?

Yes

No

Do you wash hands with soap and water after contact with animals?

Yes

No

Do you walk outside without footwear?

Yes

No

Have you received health education on environmental protection?

Yes

No

Have you participated in activities promoting environmental protection?

Yes

No
